# Evaluating antibacterial and anticancer activity of crude extracts of bacterial endophytes from *Crinum macowanii* Baker bulbs

**DOI:** 10.1002/mbo3.914

**Published:** 2019-08-17

**Authors:** Tendani E. Sebola, Nkemdinma C. Uche‐Okereafor, Kudzanai I. Tapfuma, Lukhanyo Mekuto, Ezekiel Green, Vuyo Mavumengwana

**Affiliations:** ^1^ Department of Biotechnology and Food Technology, Faculty of Science University of Johannesburg Johannesburg South Africa; ^2^ Department of Chemical Engineering, Faculty of Engineering and the Built Environment University of Johannesburg Johannesburg South Africa; ^3^ Division of Molecular Biology and Human Genetics, Department of Medicine and Health Sciences, South African Medical Research Council Centre for Tuberculosis Research Stellenbosch University Cape Town South Africa

**Keywords:** antibacterial activity, anticancer activity, *Crinum macowanii* bulbs, crude extracts, endophytes

## Abstract

The results from this study revealed that crude extracts isolated from bacterial endophytes obtained from *Crinum macowanii* bulbs showed activity against both Gram‐positive and Gram‐negative pathogenic bacteria, while *Acinetobacter guillouiae* crude extracts displayed anticancer activity. This study aimed to isolate and characterize bacterial endophytes and their crude extracts from *C. macowanii* bulbs. Endophytes were isolated using validated surface sterilization techniques, followed by phenotypic and genotypic profiles of the isolates. Crude extracts were extracted from the endophytes using ethyl acetate, while methanol:dichloromethane (1:1) was used to obtain crude extracts from the bulbs. Antibacterial activity of crude extract from each endophyte was investigated against selected pathogenic strains using the broth microdilution method, and anticancer activity against U87MG glioblastoma and A549 lung carcinoma cells was determined by the MTS (3‐(4,5‐dimethylthiazol‐2‐yl)‐5‐(3‐carboxymethoxy‐phenyl)‐2‐(4‐sulfophenyl)‐2H‐tetrazolium) assay. *Acinetobacter guillouiae*, *Pseudomonas moraviensis*, *Pseudomonas* sp., *Rahnella aquatilis*, *Bacillus cereus*, *Novosphingobium* sp., *Raoultella ornithinolytica*, and *Burkholderia tropica* were successfully isolated. The crude extracts from the majority of endophytes showed antibacterial activity, ranging from 0.125 to >16.00 mg/ml against Gram‐negative and Gram‐positive pathogenic bacteria. *Acinetobacter guillouiae* extracts showed a high bioactive potential against U87MG glioblastoma cell lines by reducing their growth by 50% at concentrations of 12.5, 6.25, and 3.13 µg/ml. Crude extracts isolated from *C*.* macowanii* bulbs showed potential for possible drug lead against common pathogenic bacteria.

## Graphical abstract

In this study, eight bacterial endophytes were isolated and crude extracts subsequently extracted. The crude extracts displayed activity against both Gram‐negative and Gram‐positive pathogenic bacteria. *Bacillus cereus* crude extracts displayed notable activity on A549 lung carcinoma cells, whereas insignificant activity was observed for all the crude extracts tested on U87MG glioblastoma cells.

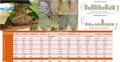

## 1 INTRODUCTION

*Crinum macowanii* Baker, from the *Amaryllidaceae* family, is a bulbous plant which has been used traditionally to treat a number of different ailments (Watt & Breyer‐Brandwijk, 1962). The bulb is used to treat itchy rashes, boils, acne, backache, venereal disease, inflamed sores, swelling of the body, urinary tract problems as well as to increase lactation in women and cows (Maroyi, 2016). Maroyi (2016) reported that *C*.* macowanii* has antimicrobial, antifungal, antiviral, and antiplasmodial properties. Although there are a number of studies validating the antimicrobial properties of *Crinum* species (Maroyi, 2016), overharvesting and overuse of medicinal plants such as *C*.* macowanii* has led to overexploitation and extinction of some of these plants (Wyk & Prinsloo, 2018). Strategies need to be deployed to find alternative methods of extracting secondary metabolites from these plants, such as using microbial sources (Chen et al., 2016; Monakisi, Esler, & Ward, 2007). An extensive literature search suggests that *C. macowanii* remains unexplored with regard to both its endophytes and the bioactivities of its crude extracts, including crude extracts derived from the bulb of this plant.

Endophytes are considered to be outstanding sources of bioactive natural compounds, as they can mimic the chemistry of their respective host plants and biosynthesize almost identical bioactive natural products, or even derivatives which can be more bioactive than those of their respective hosts (Rodriguez, White, Arnold, & Redman, 2009). As such, there has been an increase in the bioprospecting of novel efficacious bioactive compounds from microorganisms such as endophytes, to obtain novel bioactive products (Kusari & Spiteller, 2011; Martinez‐klimova, Rodríguez‐peña, & Sánchez, 2017). Bioactive secondary metabolites obtained in this way could be alternative sources of therapeutic compounds which could help eradicate problematic infections affecting the human population, for example, antibiotic resistance (Menpara & Chanda, 2013; Tidke et al., 2017).

While fungal endophytes are a source of attention in most studies, bacterial endophytes are less explored due to the small yield recoveries of crude extracts (Brader et al., 2014). Ek‐Ramos et al. (2019) has reported that metabolites produced by endophytic microorganisms' act as antimicrobial and anticancer agents against human, animal, and plant pathogens and display significant potential in medical and veterinary treatments. With this in mind, the main aim of this study was to isolate and identify bacterial endophytes from *C. macowanii* bulbs and to explore the role of endophytic crude extracts as potential antibacterial and anticancer therapeutic agents.

## 2 MATERIALS AND METHODS

### 2.1 Sample collection

Fresh, healthy *C*.* macowanii* bulbs showing no apparent symptoms of disease or herbivore damage were collected from the Walter Sisulu National Botanical Garden (Roodepoort, Gauteng, South Africa, 26°05′10.4″S 27°50′41.5″E). After collection, the samples were placed in sterile polyethylene bags and transferred to the laboratory at 4°C before being thoroughly washed with sterile distilled water and used within hours of harvesting.

### 2.2 Isolation of endophytic bacteria

The bulbs were surface sterilized separately using the method described by Jasim, Joseph, John, Mathew, and Radhakrishnan (2014) with slight modifications. Briefly, each bulb (approximately 10 g) was treated with 5% Tween‐20 (Sigma‐Aldrich) (enough to cover the plant material) and vigorously shaken for 5 min. Tween‐20 was removed by rinsing several times with sterile distilled water, followed by disinfection with 50 ml of 70% ethanol for 1 min. Traces of the ethanol were removed by rinsing with sterile distilled water 5 times. The sample was then treated with 1% sodium hypochlorite (NaClO) for 10 min and again rinsed five times with sterile distilled water. The last rinse was used as a control, and 100 µl of this was plated on potato dextrose agar (PDA; HiMedia) and nutrient agar (NA; Oxoid). The sample was then macerated in sterilized phosphate‐buffered saline (PBS), with the outer surface trimmed out. The macerated sample was serially diluted up to 10^–3^ dilution, and each dilution inoculated (using a spread plate method) in triplicate on nutrient agar. The NA plates were incubated at 30°C (IncoTherm, Labotec). Growth was monitored periodically for 5 days. Effectiveness of the sterilization was monitored on the wash control plate, with growth indicating poor sterilization. Under such circumstances, the plates for the plant part were discarded and the sterilization repeated. Distinct colonies were selected and subcultured on nutrient agar to obtain pure isolates. Pure bacterial isolates were preserved in 50% glycerol in a ratio of 500 µl glycerol:500 µl overnight broth culture and kept at −80°C.

### 2.3 Morphological identification of endophytic bacteria

#### 2.3.1 Gram staining

Pure colonies were subjected to Gram staining to establish morphological characteristics such as shape and Gram stain reaction. Gram stain slides were observed using a compound bright‐field microscope (OLYMPUS CH20BIMF200) with 1,000× magnification.

### 2.4 Molecular identification

#### 2.4.1 Genomic DNA extraction, polymerase chain reaction, and sequencing

DNA extraction was done using a ZR Fungal/Bacterial Kit™ (Zymo Research, catalog no. R2014) according to the manufacturer's instructions. Polymerase chain reaction (PCR) was done to amplify the 16S rRNA gene of each bacterial endophyte with the primers 16S‐27F: 5′‐AGAGTTTGATCMTGGCTCAG‐3′ and 16S‐1492R: 5′‐CGGTTACCTTGTTACGACTT‐3′, using DreamTaq™ DNA polymerase (Thermo Scientific™). PCR products were gel extracted (Zymo Research, Zymoclean™ Gel DNA Recovery Kit), and sequenced in the forward and reverse directions on the ABI PRISM™ 3500xl Genetic Analyser. The sequencing was performed at Inqaba Biotechnical Industries (Pty) Ltd. The PCR products were cleaned with ExoSAP‐it™ following the manufacturer's recommendations. Purified sequencing products (Zymo Research, ZR‐96 DNA Sequencing Clean‐up Kit™) were analyzed using CLC Main Workbench 7, followed by a BLAST search (NCBI) (Kuklinsky‐Sobral, Arajo, Mendes, Pizzirani‐Kleiner, & Azevedo, 2005).

### 2.5 Phylogenetic analysis

The obtained sequences were screened for chimeras using DECIPHER23 and subjected to BLAST analysis using the National Center for Biotechnology Information (NCBI) database against the 16S rDNA sequence database (bacteria and archaea) to identify the closest bacterial species. Bacterial species with 98%–100% similarities were selected for phylogenetic analysis. Alignments of nucleotide sequences were performed using MUSCLE with default options. The positions containing gaps or missing nucleotide data were eliminated. Phylogenetic trees were constructed using a neighbor‐joining (NJ) method (Saitou & Nei, 1987) based on the Tamura–Nei model (Tamura, Stecher, Peterson, Filipski, & Kumar, 2013). A total of 1,000 replications were used for bootstrap testing. All branches with greater than 50% bootstraps were considered to be significant (Soltis & Soltis, 2003). All evolutionary analyses were conducted in MEGA 7.0 (Kumar, Stecher, & Tamura, 2016). The 16S rRNA gene sequences of bacterial isolates identified in the study were deposited in GenBank (https://www.ncbi.nlm.nih.gov/genbank/) with the accession numbers as stated in Table 1. The assigned names of the bacterial isolates were based on the BLAST homology percentages as well as the phylogenetic results.

**Table 1:** The identities and morphological characteristics of the isolated bacterial endophytes from *Crinum acowanii* bulbs

Sample code	Assigned bacterial name	GenBank accession number	Similarity (%)	Gram reaction	Colony morphology (pigmentation, texture, form)
TES 01C	*Acinetobacter guillouiae*	MF943224	99	− rod	Faint yellow, viscid, circular
TES 01E	*Pseudomonas moraviensis*	MF943225	100	− rod	Faint yellow, mucoid, circular
TES 01F	*Pseudomonas* sp.	MF943226	88	− rod	Cream white, mucoid, circular
TES 03A	*Rahnella aquatilis*	MF943229	99	− rod	Cream white, pasty, rhizoid
TES 03B	*Bacillus cereus*	MF943230	99	+ rod	Cream white, moist, rhizoid
TES 03C	*Bacillus cereus*	MF943231	99	+ rod	Cream white, moist, rhizoid
TES 04A	*Novosphingobium* sp.	MF943232	99	− rod	Cream white, viscid, circular
TES 09B	*Rahnella aquatilis*	MF943238	99	− rod	Pale yellow, viscid, circular
TES 11A	*Raoultella ornithinolytica*	MF943240	99	− rod	Cream white, mucoid, filamentous
TES 12A	*Burkholderia tropica*	MF943241	98	− rods	Pale yellow, viscid, circular
TES 13A	*Rahnella aquatilis*	MF943242	100	− rods	Pale white, rugose, filamentous

### 2.6 Extraction of crude extracts from *C*.* macowanii* bulbs

*Crinum macowanii* bulbs were washed, chopped into small pieces, and air‐dried at room temperature. The dried plant material was blended into a fine powder using a commercial blender. Crude extracts were obtained according to Yadav and Agarwala (2011). Briefly, 150 g of the prepared plant material was mixed with 2 L of a 50:50 methanol:dichloromethane solution. This was allowed to shake for 3 days on a platform shaker (Amerex Gyromax) at 200 rcf. The solution was filtered through Whatman No. 1 filter paper, and the filtrate was evaporated on a rotatory evaporator and allowed to air‐dry in a desiccator.

### 2.7 Extraction of crude extracts from bacterial endophytes

For each endophytic bacterium listed in Table 1, 2 L of broth was measured into a 4‐L Erlenmeyer flask leaving room for aeration and autoclaved at 121°C for 15 min. Each 4‐L flask was inoculated with one of the endophytic bacterium as listed in Table 1 and shaken at 200 rcf and incubated at 30°C, an ideal temperature for the growth of the endophytes (Sardul Singh, Suneel, & Aharwal, 2014). After 7 days of cultivation, sterile XAD‐7‐HP resin (20 g/L; Sigma, BCBR6696V) was added to the culture for 2 hr, shaking at 200 rcf. The resin was filtered through cheesecloth and washed three times with 300 ml of acetone for each wash. The acetone‐soluble fraction was concentrated using a rotary evaporator, and a dark yellowish viscous extract was obtained, which was transferred into a measuring cylinder. Depending on the volume, ethyl acetate was added in a ratio of 1:1 (v/v). The mixture was vigorously shaken for about 10 min, decanted into a separating funnel, allowed to separate and each phase collected in a conical flask. This process was repeated until the dark yellowish viscous liquid obtained after removing the acetone became a very light‐yellow liquid. The ethyl acetate fraction was evaporated using a rotary evaporator, and the brown extract obtained was stored in an amber bottle in a cool dry place until analysis was done. The light‐yellow liquid was evaporated, and no reasonable extract or further analysis was done on this substance (Maloney et al., 2009). The brown crude secondary metabolite extracts were used for antibacterial and anticancer assays.

### 2.8 Antibacterial analysis of *Crinum macowanii* bulb crude extracts and endophytic bacterial crude secondary metabolite extracts

Microserial dilution was used to check for the minimum inhibition concentration (MIC) of the samples to specific pathogenic bacterial species, namely *Bacillus cereus* (ATCC10876), *Bacillus subtilis* (ATCC19659), *Streptococcus epidermidis* (ATCC14990), *Staphylococcus aureus* (ATCC25923), *Mycobacterium smegmatis* (ATCC21293), *Mycobacterium marinum* (ATCC927), *Enterobacter aerogenes* (ATTC13048), *Escherichia coli* (ATCC10536), *Klebsiella pneumonia* (ATCC10031), *Proteus vulgaris* (ATCC 33420), and *Proteus aeruginosa* (ATCC10145). This was done following a method described by Andrews (2001) and Sebola, Ndinteh, Niemann, and Mavumengwana (2016). The antibiotic streptomycin was used as the positive control and was prepared by weighing 0.032 mg in 1 ml of sterile distilled water while 0.1% DMSO was used as a negative control.

#### 2.8.1 Sample preparation

The crude bulb extract and crude endophytic extracts were weighed separately into empty autoclaved McCartney bottles to ensure sterility. A minimal amount of dimethyl sulfoxide (DMSO; 0.1%) was used to dissolve the crude extracts, and Mueller–Hinton (MH) broth was added to bring the volume of the dissolved crude extract to a concentration of 32 mg/ml as the stock solution.

#### 2.8.2 Microtiter plate assay

Serial dilutions were carried out using the MH broth from 16 mg/ml down to 0.031 mg/ml, which was the lowest inhibition observed. The experiment was carried out in five repeats using a 96‐well microtiter plate. The outer wells of the plate were filled with sterile distilled water (sdH_2_O). The inoculum (100 µl) was added into each well that did not contain the sdH_2_O. The diluted crude extract samples (100 µl) were added in five wells horizontally and the concentrations decreased in vertical order from 16 mg/ml down to 0.031 mg/ml. The plates were covered and incubated overnight at 37°C. After incubation, 10 µl of 0.02% (w/v) resazurin sodium salt dye solution was added to the wells and the resulting solution incubated for another 2 hr. On reduction, resazurin changes color from blue to pink to clear as oxygen becomes limited within the medium, indicating metabolism and the viability of bacterial cells, as well as no effect of the crude extracts on the bacteria. Any well with a known concentration showing a slight color change was used as MIC. The wells were visually inspected for color changes.

### 2.9 Anticancer assays

Stock solution of 200 µg/ml of all crude extracts (bulb crude extracts and endophytic crude extract) was prepared in 0.1% DMSO and sonicated. Serial dilutions were done according to McCauley, Zivanovic, and Skropeta (2013) and Artun et al. (2016). Briefly, dilutions were carried out using growth media from 100 µg/ml to 3.13 µg/ml. MTS (3‐(4, 5‐dimethylthiazol‐2‐yl)‐5‐(3‐carboxymethoxy‐phenyl)‐2‐(4‐sulfophenyl)‐2H‐tetrazolium) in vitro cancer cytotoxicity assay was carried out to determine a change in cell viability through the use of a color change. The MTS compound (yellow) is metabolized by viable cells to form a dark purple‐colored compound, while dead cells turn the color of the MTS compound pink. The samples were run in duplicate across three plates (*n* = 6), and the average values obtained were reported. The U87MG (glioblastoma) cells and A549 (lung carcinoma) cells were grown using normal tissue culture techniques using Dulbecco's modified Eagle medium (Merk) supplemented with 15% fetal bovine serum (FBS; Merck). The cells (1 × 10^5^ cells/ml) were incubated in 96‐well plates at 37°C overnight, with the subsequent addition of the crude bulb extracts and crude endophytic extracts, in concentrations of 100 μg/ml, 50.0 μg/ml, 25.0 μg/ml, 12.5 μg/ml, 6.25 μg/ml, 3.125 μg/ml, and 0 μg/ml. The cells were left to incubate for 4 days, whereupon MTS (5 μl; Promega) was added to the cells. The absorbance values were measured at 490 nm after 1‐hr, 2‐hr, and 4‐hr incubation periods. Cell viability was then calculated using the formula.

$$\%\;\;\text{Cell}\;\;\text{viability}=\left(E_{\text{a}}-B_{\text{a}}\right)/\left(C_{\text{a}}-B_{\text{a}}\right)\times 100$$

where *E*_a_ is absorbance of the extract, *B*_a_ is absorbance of the blank, and *C*_a_ is the absorbance of the control (Handayani et al., 2018). The positive control used for all conducted tests was auranofin, as it is able to inhibit thioredoxin reductase as well as the ubiquitin–proteasome system (UPS) by targeting proteasome‐associated deubiquitinase, thus inducing lung cancer cell apoptosis by selenocystine (Coussens et al., 2017; Fan et al., 2014; Roder & Thomson, 2015).

## 3 RESULTS

### 3.1 Isolation of endophytic bacteria

From the *C*.* macowanii* bulbs, a total of eleven endophytic bacteria were isolated. As the control plates did not reveal any bacterial growth, it was concluded that the isolates reported were endophytes to the plant under study. Table 1 shows the sample code, assigned bacterial name, accession number as given by GenBank, the similarity percentage between the sample isolate, the Gram stain reaction and colony morphology (color, shape, elevation, and margin) as observed on an agar plate for each endophyte.

### 3.2 Phylogenetic analysis

**Figure 1:**
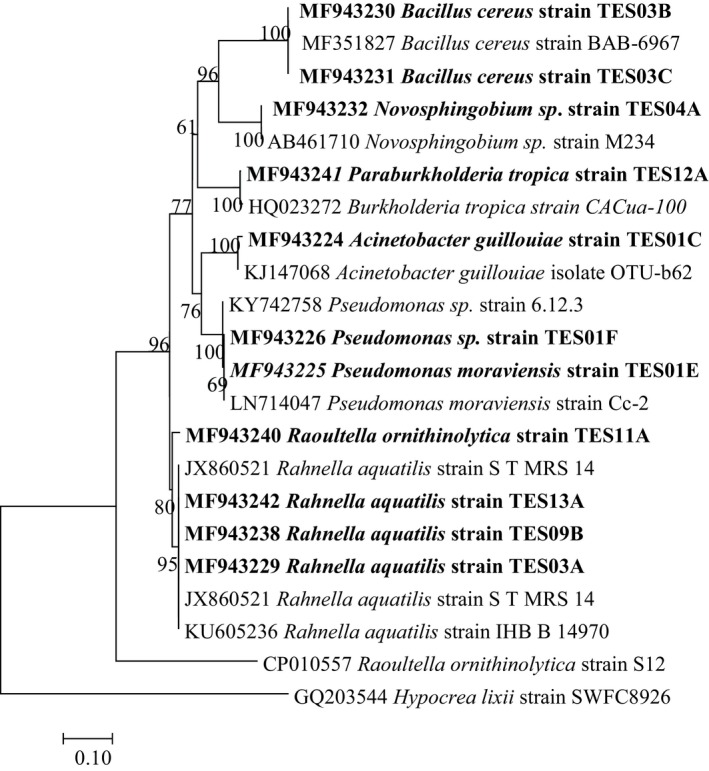
Neighbor‐joining tree based on 16S rRNA gene sequence of eleven endophytic bacteria isolated from *Crinum macowanii* bulbs and other similar species selected from GenBank

The BLAST search of the 16S rRNA gene sequences resulted in varying bacterial genera; the isolates were classified as seven genera, namely *Acinetobacter*, *Pseudomonas*, *Rahnella*, *Bacillus*, *Novosphingobium*, *Raoultella*, and *Burkholderia* (Figure 1).

### 3.3 Antibacterial evaluation of *Crinum macowanii* bulb and bacterial endophyte crude extracts

The lowest MIC (0.125 mg/ml) was observed mostly from *Pseudomonas* sp. crude extract against *B*.* subtilis*, *S*.* epidermidis*, and *M*. *marinum*. The crude extracts of most of the endophytes showed MIC values higher than 0.125 mg/ml (Table 2). The bulb extracts showed a comparable MIC only against *S*. *epidermidis*.

**Table 2:** Antibacterial evaluation of *Crinum macowanii* crude bulb extract and endophytic‐derived crude extracts

Crude extract	*B. cereus*	*B. subtilis*	*S. epidermidis*	*S. aureus*	*M. smegmatis*	*M. marinum*	*E. aerogenes*	*E. coli*	*K. pneumonia*	*P. vulgaris*	*P. aeruginosa*
Test organism with MIC (mg/ml)											
T1	0.500	16.00	0.125	8.00	0.500	0.250	16.00	8.00	>16.00	>16.00	>16.00
T2	8.00	1.00	4.00	16.00	1.00	0.500	1.00	0.500	0.500	>16.00	0.250
T3	4.00	4.00	16.00	8.00	4.00	16.00	16.00	8.00	16.00	8.00	16.00
T4	0.250	2.00	0.500	1.00	8.00	0.125	0.500	8.00	6.00	4.00	>16.00
T5	0.500	0.125	0.125	0.500	8.00	0.125	16.00	>16.00	4.00	16.00	8.00
T6	1.00	2.00	4.00	0.125	4.00	>16.00	2.00	8.00	2.00	>16.00	4.00
T7	>16.00	4.00	8.00	16.00	4.00	>16.00	16.00	>16.00	>16.00	8.00	1.00
T8	1.00	>16.00	16.00	8.00	8.00	>16.00	4.00	2.00	1.00	4.00	16.00
T9	0.500	0.250	4.00	2.00	16.00	>16.00	8.00	>16.00	>16.00	4.00	2.00
Positive control MIC (µg/ml)											
T10	0.031	0.031	0.062	0.031	0.062	0.062	0.125	0.125	0.125	0.062	0.031

Note

T1 = *C*. *macowanii* bulbs, T2 = *Raoultella ornithinolytica*, T3 = *Acinetobacter guillouiae*, T4 = *Pseudomonas moraviensis*, T5 = *Pseudomonas* sp., T6 = *Rahnella aquatilis*, T7 = *Novosphingobium* sp., T8 = *Bacillus cereus*, T9 = *Burkholderia tropica*, T10 = positive control streptomycin.

### 3.4 Anticancer assays

**Figure 2:**
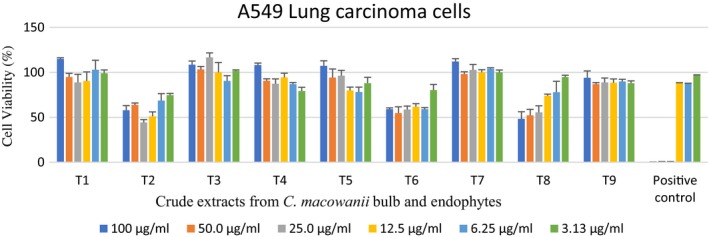
Cytotoxic activity of endophytic‐derived crude extracts and bulb crude extracts on A549 lung carcinoma cells tested at different concentrations ranging from 100 to 3.13 µg/ml. The positive control used was auranofin. T1 = *Crinum macowanii* bulbs, T2 = *Raoultella ornithinolytica*, T3 = *Acinetobacter guillouiae*, T4 = *Pseudomonas moraviensis*, T5 = *Pseudomonas* sp., T6 = *Rahnella aquatilis*, T7 = *Novosphingobium* sp., T8 = *Bacillus cereus*, T9 = *Burkholderia tropica*

Endophytic crude extracts showed varying activities against A549 lung carcinoma cells. However, T2 crude extracts showed a 62% reduction of lung carcinoma cells at a concentration of 25 µg/ml (Figure 2).

**Figure 3:**
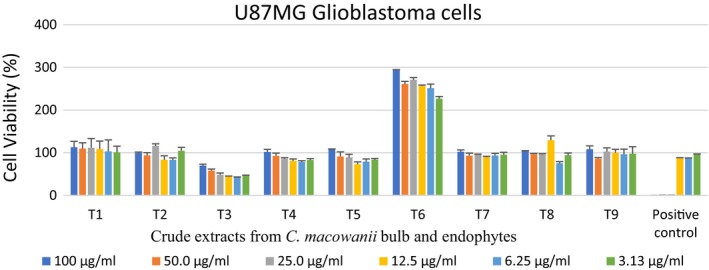
Cytotoxic activity of endophytic‐derived crude extracts and bulb crude extracts on UMG87 glioblastoma cells tested at different concentrations ranging from 100 to 3.13 µg/ml. The positive control used was auranofin. T1 = *Crinum macowanii* bulbs, T2 = *Raoultella ornithinolytica*, T3 = *Acinetobacter guillouiae*, T4 = *Pseudomonas moraviensis*, T5 = *Pseudomonas* sp., T6 = *Rahnella aquatilis*, T7 = *Novosphingobium* sp., T8 = *Bacillus cereus*, T9 = *Burkholderia tropica*

*Acinetobacter guillouiae* extract T3 also showed promising activity when tested against brain cancer cell lines, with the highest concentration, (100 µg/ml), showing minimal activity while at 6.25 µg/ml concentration displayed a 50% reduction in the cell viability of brain cancer, compared to the other extracts (excluding extract T6; Figure 3).

## 4 DISCUSSION

Endophytic bacteria are the most unexplored yet diverse group of microorganisms with a symbiotic association with plants, and are promising sources of biologically active agents (Raghu, 2012). Very few plants have been explored for their endophytic variety as most are found in distinct biological niches (Shiva Kameshwari, Mohana, & Thara Saraswathi, 2015).

Eleven bacterial strains were isolated from *C. macowanii* in this study. Micro‐morphological analyses of the culturable isolates showed that nine were Gram‐negative rods while two were Gram‐positive rods. Ullah et al. (2018) reported that roots and bulbs harbor a greater number of endophytes with a diverse population, as compared to other plant parts of the plant. Menpara and Chanda (2013) and Rhoden, Garcia, Santos e Silva, Azevedo, and Pamphile (2015) reported that *Pseudomonas*, *Acinetobacter*, *Staphylococcus*, *Bacillus*, *Burkholderia*, *Enterobacter*, *Pantoea*, and *Agrobacterium* are the most predominant endophytes in medicinal plants.

The use of 16S rDNA gene sequence revealed that the isolated endophytes belong to diverse bacterial groups, namely the genera *Pseudomonas*, *Bacillus*, *Acinetobacter*, *Rahnella*, *Novosphingobium*, *Raoultella*, and *Burkholderia.* Our results support the findings of Menpara and Chanda (2013) and Rhoden et al. (2015), who found *Bacillus* and *Pseudomonas* to be the most frequently isolated endophytes in medicinal plants. Sequence identity of two of our isolates (TES03B and TES03C) showed 100% similarity to *B. cereus* BAB‐6967, while TES01E and TES01F were similar to the *Pseudomonas* strain 6.12.3. *Burkholderia tropica* endophytes have been useful in nitrogen fixation and thus increase plant nutrient availability (Tenorio‐salgado, Tinoco, Vazquez‐duhalt, Caballero‐mellado, & Perez‐rueda, 2013). Mercado‐blanco and Lugtenberg (2014) reported that *Rahnella aquaqtilis* endophytes stimulate plant growth of cereals and radishes, whereas endophytic *Pseudomonas* spp. have been used as biocontrols of different phytopathogens (Mercado‐blanco & Lugtenberg, 2014). Endophytic *Novosphingobium* sp. has been reported to grow in rice plants and promotes the growth of rice (Rangjaroen et al., 2017). To the best of our knowledge, *A. guillouiae*, *Novosphingobium* sp., *B. tropica*, and *R. aquaqtilis* have not been reported in *C*. *macowanii* bulbs prior to this study.

*Crinum macowanii* bulbs have been used to treat diseases or ailments caused by bacteria (Maroyi, 2016). Rabe and Van Staden (1997) tested methanol extracts of *C. macowanii* bulbs on a number of bacteria such as *E*. *coli*, *K*. *pneumoniae*, *S. aureus*, and *S*. *epidermidis* and observed no antibacterial activities. Sebola et al. (2016) reported the inhibition of *B*. *cereus*, *M*. *smegmatis*, and *S*. *epidermidis* at 0.5 mg/ml, 0.125 mg/ml, and 0.0625 mg/ml, respectively, from methanol/dichloromethane (1:1, v/v) crude extracts of the bulb. The results obtained in this study indicate the inhibition of *B. cereus*, *S. epidermidis*, and *M. smegmatis* at 0.500 mg/ml, 0.125 mg/ml, and 0.500 mg/ml, respectively, which concur with the previous study of Sebola et al. (2016). Inhibition greater than 16 mg/ml, observed in *K*. *pneumonia*, *P*. *vulgaris*, and *P*. *aeruginosa*, was not considered to be inhibitory. The inhibition of other bacteria by *C*. *macowanii* bulb crude extracts could be due to the relationship between the plant and its endophytes, which appears to confer certain benefits such as increased resistance to disease and induced growth (Rodriguez et al., 2009).

Endophytic bacteria have been reported to produce a number of secondary metabolites such as alkaloids, steroids, terpenoids, peptides, and flavonoids with antibacterial, antifungal, and cytotoxic properties (Raghu, 2012). The endophytic crude extracts tested in this study showed antibacterial activity against selected pathogenic strains. *Raoultella ornithinolytica* crude extract had MIC values ranging from 0.250 to 16 mg/ml, with the most significant inhibition observed for *K. pneumonia*, *E. coli*, and *P.* *aeruginosa* at concentrations of 0.500 mg/ml, 0.500 mg/ml, and 0.250 mg/ml, respectively. To the best of our knowledge, this study is the first to report on the extraction of crude extract from *R. ornithinolytica*, *A. guillouiae*, *Rahnella aquatilis*, and *Novosphingobium* sp. endophytes and their antibacterial activity. The results show promising antibacterial activity, as crude extracts of activity <1 mg/ml are deemed to have noteworthy antibacterial properties (Zonyane, Makunga, & Vuuren, 2013). Crude extracts from *B. cereus* showed MIC values of between 1 and 16 mg/ml, inhibiting *K. pneumonia* at 1 mg/ml. Crude extract from *B. cereus* has been reported to possess antibacterial activity against a wide range of pathogenic microbes such as *E*. *coli* and *K*. *pneumoniae* (Kumar, Thippeswamy, & Shivakumar, 2013). This supports the findings in this study.

*Pseudomonas moraviensis* crude extract showed MIC values of between 0.125 and >16 mg/ml. The crude extract showed activity against *B. cereus*, *S*. *epidermidis*, *S*. *aureus*, *M*. *marinum*, and *E*. *aerogenes* at concentrations of 0.250 mg/ml, 0.500 mg/ml, 1.00 mg/ml, 0.125 g/ml, and 0.500 mg/ml, respectively. *Pseudomonas moraviensis* crude extract had no significant activity on *P. aeruginosa*, showing values of >16 mg/ml (highest tested concentration). *Pseudomonas moraviensis* has been reported to possess antibacterial proteins and peptides such as bacteriocins (Ghequire & De Mot, 2014). *Pseudomonas* spp. have a significant metabolic potential due to genetic loci encoding secondary metabolites (Davis, 2017; Gross & Loper, 2009; Silby, Winstanley, Godfrey, Levy, & Jackson, 2011). Wauven et al. (2014) and Mohamed, Avis, and Tsopmo (2015) reported that *Pseudomonas* sp. produced antimicrobials such as mupirocin, pyrrolnitrin, and pyoluteorin. This could justify the results we observed. This may suggest that extracts from *P. moraviensis* could be used as antibacterial agents.

*Rahnella aquatilis* crude extract had MIC values ranging between 0.125 and >16 mg/ml. *Staphylococcus aureus* was inhibited at 0.125 mg/ml. El‐Hendawy, Osman, and Sorour (2003) reported that *R. aquatilis* strains obtained from soil were able to produce bacteriocin, which inhibited culture of different Gram‐positive and Gram‐negative bacteria. *Novosphingobium* sp. crude extract had MIC values ranging from 1 to >16 mg/ml, showing a lack of activity against the tested organisms. *Burkholderia tropica* crude extract had MIC values ranging from 0.250 to >16 mg/ml. *Bacillus subtilis* was inhibited at 0.250 mg/ml. *Mycobacterium marinum*, *E. coli*, and *K. pneumonia* had MIC values of >16 mg/ml, which was deemed noninhibitory. *Burkholderia* showing biocontrol and plant growth‐promoting characteristics was reported (Ho & Huanga, 2015). It was observed in this study that the Gram‐positive bacterial species were more susceptible to the antibacterial compounds in the crude extracts than the Gram‐negative bacteria. This could be attributed to the difference in the cell walls of both groups of bacteria, as Gram‐negative bacteria are known to be resistant to most antibiotics due to their outer membrane, which tends to expel antibiotics from the cells by acting as a selective barrier (in contrast to that of their Gram‐positive counterparts) (Delcour, 2009; Iannello et al., 2014).

Greenwell and Rahman (2015), Seca and Pinto (2018) reported that secondary metabolites (alkaloids, brassinosteroids, and taxols) from plants possess anticancer properties, and most are currently in clinical trials or being used in therapeutic applications. *Crinum macowanii* bulb crude extract T18 showed a cell viability of above 80% against A549 lung carcinoma, and an above 100% cell viability was observed on UMG87 glioblastoma cell lines for all the concentrations tested, indicating no activity on those cell lines. In contrast, Maroyi (2016) reported that alkaloids such as crinamine, bulbispermine, and lycorine isolated from *C*. *macowanii* have cytotoxic activity against human oral epidermoid carcinoma KB cells, apoptosis‐resistant cell lines, and BLS mouse melanoma cells.

*Acinetobacter guillouiae* crude extract showed notable activity on UMG87 glioblastoma cell lines, with 58% cell death at 6.25 µg/ml. However, increased concentrations of *A. guillouiae* crude extract showed an increase in cell viability. The extracts had a lower cell viability compared to that of the positive control at this concentration. *Acinetobacter* sp. are known to contain amine compounds reported to have higher tumor toxicity against human oral squamous cell carcinoma (Arora, 2015; Shimada et al., 2014). Those amines could be responsible for the activity observed. *Bacillus cereus* crude extract exhibited a low cell viability of 48% at 100 µg/ml on the A549 lung carcinoma cell lines. Other species of *B. cereus* have been reported to have cytotoxicity on a human cervical cancer cell line (HeLa) and a breast cancer cell line (MCF‐7) (Ferdous, Shishir, Khan, & Hoq, 2018). This could explain the results obtained in this study. *Raoultella ornithinolytica* crude extract exhibited a low cell viability of 57% at 100 µg/ml on the A549 lung carcinoma cell lines, despite a recovery of cell viability when the cells were exposed to a low concentration of 3.13 µg/ml. This observation is supported by studies carried out by Zhang et al. (2006) where the researchers reported that after a low dose of chemotherapy, tumor tissue has a propensity to regrow, causing tumor repopulation. Hutf and Grady (1996) stated that anticancer drugs used for lung carcinoma cells have concentration–effect relationships and this could explain our results.

A puzzling finding was the increased cell viability in the UMG87 glioblastoma cell in response to the *R. aquatilis* crude extract. However, UMG87 glioblastoma cell lines are known to undergo hypoxia, resulting in metabolic abnormalities such as increased uptake of glucose and acid resistance; this increased glucose uptake and high aerobic glycolysis induces proliferation of cancer cells (Jiang, 2017; Zhou et al., 2011). This would explain the high cell viability of 100% and above on the glioblastoma cell lines. Further studies are needed to elucidate this phenomenon. The activities observed from the methanol/dichloromethane crude plant extracts could be caused by artifacts as Sauerschnig, Doppler, Bueschl, and Schuhmacher (2018) mentioned that artifacts are generated by methanol during sample extraction and storage.

## 5 CONCLUSION

A diverse microbial community was isolated from *C. macowanii* bulbs, with notable inhibitory activities against Gram‐positive and Gram‐negative bacterial species. From these results, it can be concluded that endophytic‐derived crude extracts isolated from medicinal plant *C*. *macowanii* bulbs produce potential bioactive compounds which should be explored further for their biological activities.

## CONFLICT OF INTERESTS

None declared.

## AUTHOR CONTRIBUTIONS

Tendani Sebola involved in project administration. Vuyo Mavumengwana and Tendani Sebola conceived the study. Vuyo Mavumengwana and Ezekiel Green (co‐supervisor) involved in supervision. Lukhanyo Mekuto, Vuyo Mavumengwana, and Ezekiel Green provided resources. Lukhanyo Mekuto, Ezekiel Green and Vuyo Mavumengwana funded the study. Tendani Sebola, Nkemdinma Uche‐Okereafor, and Kudzanai Tapfuma investigated the study. Tendani Sebola involved in visualization and validation. Tendani Sebola wrote the original draft of the manuscript. Tendani Sebola, Nkemdinma Uche‐Okereafor, Kudzanai Tapfuma Lukhanyo Mekuto, Vuyo Mavumengwana, and Ezekiel Green reviewed and edited the manuscript. Lukhanyo Mekuto and Vuyo Mavumengwana were involved in the acquisition of funds for isolation of endphytes and anticancer study. Ezekiel Green was invovled in the acquisition of funds for the antibacterial study.

## ETHICS STATEMENT

None required.

## Data Availability

All data are provided in full in the results section of this paper apart from the DNA sequences of the bacterial endophytes are available at https://www.ncbi.nlm.nih.gov/genbank, and accession numbers for each endophyte can be found in Table 1.
